# Associations between postpartum pain type, pain intensity and opioid use in patients with and without opioid use disorder: a cross-sectional study

**DOI:** 10.1016/j.bja.2022.09.029

**Published:** 2022-11-10

**Authors:** Grace Lim, Kelsea R. LaSorda, Elizabeth Krans, Bedda L. Rosario, Cynthia A. Wong, Steve Caritis

**Affiliations:** 1 Department of Anesthesiology and Perioperative Medicine, University of Pittsburgh School of Medicine, Pittsburgh, PA, USA; 2 Department of Obstetrics, Gynecology & Reproductive Sciences, University of Pittsburgh School of Medicine, Pittsburgh, PA, USA; 3 Center for Innovation in Pain Care, University of Pittsburgh, Pittsburgh, PA, USA; 4 Magee-Women's Research Institute, Pittsburgh, PA, USA; 5 School of Epidemiology, University of Pittsburgh, Pittsburgh, PA, USA; 6 Department of Anesthesia, Roy J. and Lucille A. Carver College of Medicine, University of Iowa, Iowa City, IA, USA

**Keywords:** affective pain, multidimensional pain, obstetric care, opioid use disorder, pain intensity, perioperative care, postpartum pain, substance use disorder

## Abstract

**Background:**

Pain is a multidimensional construct. The purpose of this cross-sectional, single-centre study was to evaluate the relationship between postpartum pain type with pain intensity and opioid use in people with and without opioid use disorder (OUD).

**Methods:**

Postpartum pain type was coded from McGill Pain Questionnaire and Patient-Reported Outcome Measurement Information System (PROMIS) inventories in people with or without OUD after childbirth in a 4-month period. The co-primary outcomes were pain intensity (0–10 scale) and total inpatient oxycodone (mg). Multivariable linear mixed-effects models assessed between- and within-person relationships for pain type (primary predictor) and outcomes.

**Results:**

There were 44 522 unique pain scores and types from 2610 people. Pain types were associated with pain intensity (*P*<0.001). Between-person comparisons showed affective pain was associated with a small but higher total oxycodone dose (difference 1.04 mg compared with no affective pain, *P*<0.001). Among people with OUD, within-person comparisons showed that the presence of affective pain resulted in pain scores 1 point higher than when affective pain was not present (*P*=0.002); between-person comparisons showed that people with affective pain had pain scores 6 points higher (*P*=0.048). Within-person and between-person comparisons among OUD showed that nociceptive/neuropathic pain was associated with a higher total oxycodone dose (1.6 and 11.4 mg, respectively).

**Conclusions:**

Postpartum pain type was associated with pain intensity and opioid use. Further research is required to address the multiple dimensions of postpartum pain in people with and without OUD to improve treatment of postpartum pain.

**Editor's key points**

• The relationship between maternal pain type, pain intensity, and inpatient opioid requirements after childbirth is unclear.
• In this cohort of 2610 people providing 44 522 unique pain ratings after childbirth, pain type was significantly associated with pain intensity and inpatient opioid use after delivery. Among people with opioid use disorder, affective and nociceptive/neuropathic pain dimensions were key drivers of pain intensity and opioid use.
• Tailored pain treatments that address nociceptive, neuropathic, and affective pain dimensions are needed to reduce pain and opioid use after birth.

Pain is a complex process, influenced by individual biology, psychology, and social factors. Multiple dimensions, including sensory, cognitive, and affective (i.e. emotional) aspects of pain, exist which can differentially influence how a patient experiences the intensity and quality of pain. In 2020, the International Association for the Study of Pain (IASP) revised the definition of pain from one that ignored the multiplicity of pain and the cognitive and social factors that are integral to its experience, to a definition which now acknowledges the multidimensional nature of pain: ‘Pain is an unpleasant sensory and emotional experience associated with, or resembling that associated with, actual or potential tissue damage’.^1^ This updated definition calls for improvements in assessment and management of pain, and emphasises the following points: pain is a personal experience; pain cannot be exclusively inferred from sensory neurone activity; life experiences and learned pathways influence the individual concepts of pain; a person's report of an experience of pain should be respected; and pain can be adaptive and maladaptive, leading to impaired function and social/psychological well-being.

Despite the well-recognised nuances of patient pain experiences, typical postpartum clinical pain assessments are currently limited to an assessment of pain intensity alone using the 11-point (0–10) numeric rating scale (NRS). Postpartum analgesia regimens have also remained rooted in a one-size-fits-all approach, often using opioids to treat moderate to severe breakthrough pain, no matter the pain type.^2^ The lack of an individualised approach to postpartum pain management may differentially affect subpopulations of patients who have more complex pain experiences, such as people with opioid use disorder (OUD). People with OUD frequently have severe post-traumatic pain, possibly because of hyperalgesia and the limited effectiveness of opioids in the presence of other systemic opioids such as buprenorphine.^3^^,^^4^ Patients with OUD are also at greater risk for adverse outcomes in the perioperative and postpartum period, including higher risk for return to use, overdose, overdose mortality, readmission, and postoperative complications than patients without OUD.^5^, ^6^, ^7^

Recent efforts have successfully curbed over-prescribing of opioids in postpartum patients using shared decision-making approaches.^8^^,^^9^ Shared decision-making seeks to integrate patients' pain experiences into opioid prescribing practices, but it is also possible that patients' fear of opioids may drive prescribing decisions that unintentionally lead to sub-optimal pain management.^10^ Past studies have focused on prescribing behaviour,^11^^,^^12^ but focusing on prescribing alone overlooks the need to also understand the impact of a multidimensional pain experience on postpartum opioid use. Poorly treated pain has multiple negative ramifications, including maladaptive social/psychological function, and in some cases, a progressive transition to chronic pain.^13^, ^14^, ^15^, ^16^ In patients with OUD, overly restrictive opioid prescribing policies could have the unintended consequence of accessing the illicit drug market for pain control.^17^ An understanding of the factors that influence postpartum pain is especially important for people with OUD who are at increased risk for return to use and overdose in the postpartum period.^6^^,^^7^

The purpose of the present study was to understand the dimensions of pain that people experience in the postpartum period, and the influence of pain type on postpartum pain intensity and opioid use. Specifically, our objectives were to: (1) examine the association of pain type on pain intensity and opioid use during the inpatient hospitalisation after delivery; and (2) evaluate whether the influence of pain type on intensity and opioid use was distinctly different for people with OUD. We hypothesised that pain types (predictors) were associated with postpartum pain intensity and postpartum opioid dose (co-primary outcomes), and secondarily, that these patterns differ among people with OUD.

## Methods

The study was approved by University of Pittsburgh Institutional Review Board (STUDY19120054) and followed the Strengthening the Reporting of Observational Studies in Epidemiology (STROBE) reporting guidelines. The requirement for informed consent for this study involving data abstracted from the medical record was waived.

The study design was a cross-sectional study of all people admitted to University of Pittsburgh Medical Center Magee for labour and delivery from March 2018 to June 2018. Data abstracted from the medical record included all pain scores and pain types prospectively reported by each patient, total inpatient opioid dose, maternal age, estimated gestational age at delivery, gravidity, parity, mode of delivery, and history or active diagnosis of OUD. At our institution, pregnant patients with OUD are managed by the Pregnancy Recovery Center clinic, which manages buprenorphine or buprenorphine–naloxone therapy, or interfaces with relevant outpatient clinics for optimisation of methadone therapy. A history or active diagnosis of OUD with or without medication for opioid use disorder (MOUD) was coded by documentation of active medications listed on hospital intake forms (i.e. methadone, buprenorphine, buprenorphine–naloxone) and International Classification of Diseases, 9th Revision (ICD-9) or -10 code for opioid use disorder or dependence (Supplemental Material 1); there were no pregnant patients with chronic pain diagnosis in the current cohort. All pain and oxycodone variables starting immediately after delivery until discharge were recorded. Data were abstracted directly from the medical records using automated electronic reporting. No data were abstracted manually.

### Postpartum clinical care and analgesia protocols

Our institutional postpartum analgesia protocols are based primarily on mode of delivery. The average length of stay after Caesarean and vaginal deliveries are 3.2 and 2.1 days, respectively. For Caesarean delivery, almost all patients receive neuraxial morphine with intrathecal doses ranging from 100 to 150 μg and epidural doses from 3 to 3.5 mg. During this study period, post-Caesarean pain management for patients not receiving neuraxial morphine differed from patients who did receive neuraxial morphine in that the former would be offered patient-controlled analgesia (typically hydromorphone) if pain was not controlled postoperatively. Patients having Caesarean delivery receive scheduled oral acetaminophen 650 mg and oral NSAIDs such as ibuprofen 600 mg, every 6 h. These non-opioid medications are available on a *pro re nata* basis for vaginal delivery patients. For patients having Caesarean deliveries, and rarely for vaginal delivery, oxycodone 5–10 mg is provided as needed for moderate or severe breakthrough pain rated 7 or higher on the NRS for pain. The bedside nurse offers the oxycodone to the patient and the patient can refuse or accept. Patients with OUD who have severe pain are offered opioids, but the decision to use opioids for pain control is typically individualised using shared decision making between the physician and patient, based on individual treatment goals for their treatment for OUD.

### Predictor: pain types

The predictor of interest was pain type. In accordance with existing clinical practice, pain experience was assessed and documented in the medical record by the bedside nurse. Pain was assessed quantitatively using the NRS score (0=no pain at all to 10=greatest pain imaginable). Per our institutional protocols, for each numeric pain rating that is given, patients were also asked to provide a qualitative pain description. The listed descriptive terms were stated by the bedside nurse with each evaluation and included: ‘ache’, ‘awful/bad’, ‘burning’, ‘cramping’, ‘crushing’, ‘contraction’, ‘coughing’, ‘heavy/pressure’, ‘movement’, ‘numb’, ‘positional’, ‘radiating’, ‘sharp/stabbing’, ‘shooting’, ‘sore’, ‘spasm’, ‘spasm’, ‘tender’, ‘throbbing’, or ‘tingling’. Any combination of these terms could be selected for each pain rating (e.g. a patient reporting 7/10 pain could describe it as both awful/bad and cramping). The bedside nurse documented these ratings in the medical record at the time of assessment. The nurse could free text a brief description if the patient provided words other than those given in the list to describe their pain. The pain scores and descriptors were assessed every 6 h for the entirety of the inpatient postpartum hospitalisation as part of routine clinical care using an existing template embedded in the electronic health record. Patients could report multiple pain descriptors and scores during a single assessment, and these ratings were logged separately. For example, the above patient reporting pain rated 7/10 and described as both awful/bad and cramping, would be logged in the medical record as ‘7 awful/bad’, and ‘7 cramping’ at that time point. Opioid use was defined as the total oxycodone dose (mg) administered during the postpartum period, typically 2 days for patients after a vaginal delivery and 3 days for patients after a Caesarean delivery.

### Pain types: descriptor codes

The patient-reported pain descriptors abstracted from the electronic health record were coded by an investigator (GL) into seven pain types using the established terms from the Short-Form McGill Pain Questionnaire (SF-MPQ)^18^ and Patient-Reported Outcome Measurement Information System (PROMIS) inventories (Nociceptive Pain Quality 5a^19^^,^^20^ and Neuropathic Pain Quality 5a^21^): affective/evaluative, visceral/nociceptive, somatic/nociceptive, dynamic/evoked, neuropathic, nociceptive, and nociceptive/neuropathic (Table 1). Pain intensity scores (0–10) were recorded with each pain descriptor. The experience of pain encompasses a sensory component (i.e. transmission of the pain signal from peripheral nerves to central nervous system), and an affective (brain processing) component.^18^ Nociceptive pain develops in response to a specific situation such as tissue injury, and changes with movement, position, or load. Neuropathic pain is caused by damage or injury to the nervous system itself, such as nerves that transfer the pain signals. The SF-MPQ and PROMIS inventories are valid and reliable tools to assess nociceptive and neuropathic. The SF-MPQ separates sensory and affective dimensions of pain, including effective discrimination for neuropathic and non-neuropathic pain conditions. The PROMIS inventories isolate neuropathic and nociceptive pain types. Dynamic and evoked pain were defined as pain provoked by movement, such as deep breathing or coughing, getting out of bed, or walking.^22^

**Table 1:** SF-MPQ/PROMIS pain categories, coded by patient-reported pain descriptors. PROMIS, Patient-Reported Outcomes Measurement Information System; SF-MPQ, Short-Form McGill Pain Questionnaire.

SF-MPQ/PROMIS Pain type	Patient-reported pain descriptor
Affective/evaluative	Awful, bad
Visceral	Ache, contraction, cramping, spasm
Somatic/nociceptive	Throbbing, crushing, heavy
Dynamic/evoked	Movement, positional, coughing
Neuropathic	Burning, numb, tingling
Nociceptive	Sore
Nociceptive/neuropathic	Tender, radiating

For the current study, patient-reported pain descriptors were abstracted from the electronic health record as recorded by the nursing assessments, described more completely in the above ‘Predictor: pain types’ section. These descriptors were then coded into seven pain types using the above established terms from the SF-MPQ and PROMIS inventories.

### Statistical analysis

The co-primary outcomes were pain intensity scores (0–10 scale) and in-patient opioid dose defined as oxycodone dose in milligrams. A conservative and Bonferroni corrected *P* = 0.025 was required for each of the primary outcomes to be considered statistically significant. Continuous data were assessed for normality using box plots and histograms. We compared the descriptive statistics for demographic and obstetric characteristics between OUD and non-OUD groups with the χ^2^ test (categorical variable) and t-tests for continuous variables. Violin plots of frequency for each pain rating (0–10 NRS) were constructed according to mode of delivery and OUD group. For descriptive statistics, the relationship between OUD status and pain scores, by Caesarean delivery and vaginal delivery, were compared by one-way analysis of variance (anova). The frequency of pain types between OUD groups according to mode of delivery (vaginal *vs* Caesarean delivery) were compared using the χ^2^ statistic. Missing data were treated as missing and only complete data (both pain scores and descriptors were reported) were analysed.

**Fig 1:**
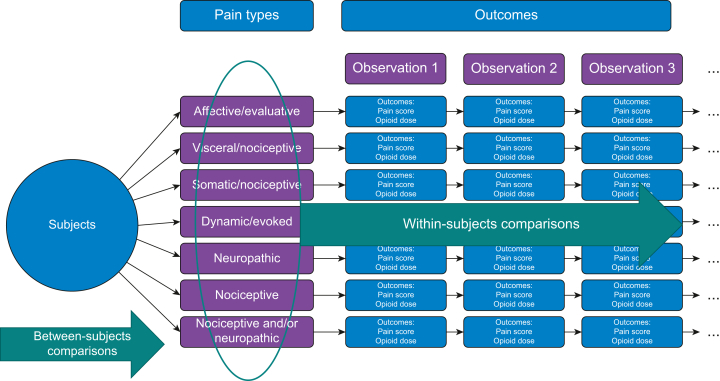
Mixed-effects conceptual model informing the primary analyses. Between- and within-subjects comparisons were made for relationships between pain type and the primary outcomes, pain score and opioid dose. For models where interaction terms were not significant, history of opioid use disorder was separately analysed for pain types as predictors; pain scores and oxycodone dose were co-primary outcomes.

For the primary analysis, to account for repeated measures from the same person (within-person comparisons) and between individuals (between-person comparisons), multivariable linear mixed-effects model analyses were performed as previously demonstrated in studies on pain and opioid consumption^23^, ^24^, ^25^ (Fig. 1). These models assessed between- and within-person relationships for the following: (1) pain type (dependent variable) and pain intensity (independent variable); and (2) pain type (dependent variable) and oxycodone dose (mg) (independent variable). These relationships were queried to answer the primary questions of whether pain intensity varies by pain type, and how oxycodone dose varies by pain type. Covariates included maternal age, gravidity, parity, estimated gestational age, mode of delivery, presence of perineal lacerations, and history of OUD. For models in which interaction terms were not significant, history of OUD was separately analysed for pain types as predictors and pain scores and oxycodone dose as outcomes. Subjects were considered random effects. In models adjusted for covariates, age, gravidity, parity, mode of delivery, and history of OUD were treated as fixed effects. We also evaluated the models for interactions between pain type and a history of OUD. For the whole cohort, opioid dose requirement was compared by pain type after adjusting for covariates. Coefficients were evaluated to assess strength of relationships between pain types and opioid dose. Where significant interactions were noted between pain type and a history of OUD, we performed separate mixed linear effects models for patients with and without OUD. All statistical analyses were performed with SAS version 9.3 (SAS Institute Inc., Cary, NC, USA) and *P*-values were two-sided with a significance level of 0.025 for the primary outcomes and 0.05 for all other analyses.

## Results

During the 4-month study period, 2610 people provided 44 522 unique pain scores and pain descriptors during their postpartum hospital stay with an average of 17.1 ratings per subject; 687 people (28.6%) had a Caesarean delivery. A history of OUD was present in 82 (3.1%) of the sample. Other demographic characteristics are presented in Table 2. The convenience sampling strategy yielded 95% confidence intervals (CIs) for mean pain intensity scores: for people without OUD, the mean pain score was 4.24 (standard deviation, 2.57; 95% CI, 4.21–4.26). For people with OUD, the mean pain score was 5.58 (standard deviation, 2.57; 95% CI, 5.47–5.69).

**Table 2:** Patient demographic, obstetric, and postpartum opioid dose characteristics. Data are reported as mean (standard deviation or range) or % [*n*]. ∗*P*<0.001 for No OUD compared with OUD. OUD, opioid use disorder.

Variable	No OUD (*n*=2528)	OUD (*n*=82)
Age (yr)	30.0 (14–44)	30.1 (20–41)
Vaginal delivery∗	56 [1428]	35 [29]
Caesarean delivery∗	44 [1100]	65 [53]
Estimated gestational age (weeks)∗	37.9 (4.1)	36.5 (5.6)
Gravidity∗		
1	33.7 [851]	17.6 [14]
2	26.9 [681]	19.0 [16]
3	17.6 [445]	14.7 [12]
4+	21.8 [551]	48.7 [40]
Parity		
0	1.4 [36]	1.4 [1]
1	44.5 [1124]	44.5 [36]
2	29.5 [746]	29.5 [24]
3	15.3 [386]	15.3 [13]
4+	9.3 [236]	9.3 [8]
Caesarean delivery oxycodone dose (mg)∗	2.9 (2.7)	4.2 (3.9)
Vaginal delivery oxycodone dose (mg)∗	1.6 (1.5)	2.9 (2.6)

### Descriptive analysis on pain types

In the whole cohort, the most commonly reported pain types were visceral (67.4%), followed by nociceptive (40.2%). Visceral pain differences by mode of delivery were noted in people with and without OUD: in people without OUD, rates of visceral pain were different between vaginal (67.8%) and Caesarean delivery (48.2%) groups (χ^2^=1.3, *P*<0.0001). Similarly, in people with OUD, rates of visceral pain were different between vaginal (63.4%) and Caesarean delivery (45.8%) groups (χ^2^=65.2, *P*<0.0001) (Table 3).

**Table 3:** Frequency of pain types compared between patients with and without OUD, by mode of delivery. OUD, opioid use disorder. *n*, total number of pain descriptors.

Caesarean delivery					
Pain descriptors	**No OUD (*n*** = **18 490)**	**%**	**OUD (*n*** = **1478)**	**%**	***P*-value**
Visceral	8903	48.2	677	45.8	0.087
Affective/evaluative	178	1.0	53	3.6	<0.001
Neuropathic	486	2.6	76	5.1	<0.001
Somatic/nociceptive	218	1.2	13	0.9	0.31
Dynamic/evoked	9	0.0	1	0.1	0.75
Nociceptive	7878	42.6	601	40.7	0.16
Nociceptive/neuropathic	818	4.4	57	3.9	0.32
**Vaginal delivery**					
**Pain descriptors**	**No OUD (*n*** = **20 161)**	**%**	**OUD (*n*** = **716)**	**%**	***P*-value**

Visceral	13665	67.8	454	63.4	0.395
Affective/evaluative	147	0.7	18	2.5	<0.001
Neuropathic	167	0.8	12	1.7	0.01
Somatic/nociceptive	235	1.2	13	1.8	0.08
Dynamic/evoked	6	0.0	1	0.1	0.1
Nociceptive	5454	27.1	200	27.9	0.19
Nociceptive/neuropathic	487	2.4	18	2.5	0.71

**Fig 2:**
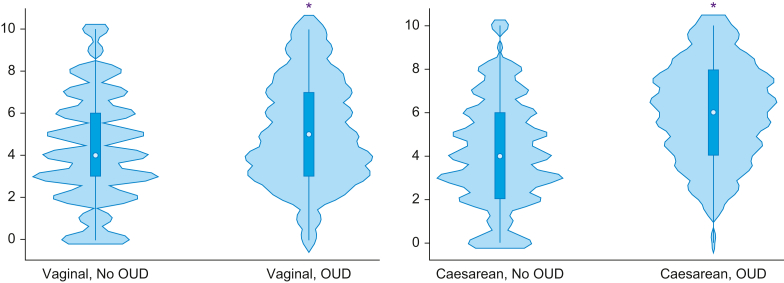
Violin plots for percent frequency for each pain rating, according to vaginal *vs* Caesarean delivery, and opioid use disorder (OUD) condition. The plots compare the distributions of pain rating data between groups; peaks, valleys, and tails of each group's density curve visualise comparisons where the groups are similar or different. For both vaginal and Caesarean deliveries, there was a statistically significant difference in pain scores between patients with and without OUD. ∗*P*<0.001.

Between OUD and no OUD groups, significant differences in pain type were noted; specifically, people with OUD had lower proportions of visceral pain (OUD 52%, no OUD 58%; *P* < 0.001), and higher proportions of affective/evaluative pain (OUD 3%, no OUD 1%; *P* < 0.001), neuropathic pain (4% *vs* 2%, *P*<0.001), and nociceptive pain (37% *vs* 34%, *P*=0.002). When comparing pain types by mode of delivery between groups, people with OUD experiencing a vaginal delivery experienced more neuropathic (no OUD 1% *vs* OUD 2%, *P* < 0.05) and affective/evaluative pain (no OUD 1% *vs* OUD 3%, *P* < 0.05) than people without OUD having vaginal delivery (Table 3). Post-Caesarean delivery pain had at significantly higher frequencies for neuropathic (no OUD 3% *vs* OUD 5%, *P*<0.001) and affective/evaluative pain (no OUD 1% *vs* OUD 4%, *P*<0.001) in patients with OUD (Table 3). Pain scores were different between OUD groups: Among people having Caesarean deliveries, there was a statistically significant difference in pain scores between people with and without OUD (*F*_1, 19 963_=349.7, *P*<0.001). Among people having vaginal deliveries, there was a statistically significant difference in pain scores between people with and without OUD (*F*_1, 24 827_=130.4, *P*<0.001). Figure 2 displays the distributions of pain scores by mode of delivery for people with and without OUD.

### Linear mixed models analysis for pain intensity scores according to pain type

Results of multivariable linear mixed model analysis for pain type with pain scores for the cohort, after adjusting for covariates, revealed key differences between people with certain pain types, and within individual people experiencing different pain at different times (see Supplemental Material 2 for expanded tables with interpretations). Within-person comparisons showed that NRS scores were 0.77 points higher at time points when people had neuropathic pain compared to times without neuropathic pain (95% CI, 0.61–0.92; *P*<0.0001) (Supplemental Material 2). Between-person comparisons showed that the pain score was 2.75 points higher for a person with neuropathic pain compared with a person without neuropathic pain, controlling for other covariates (95% CI, 2.17–4.77; *P*<0.0001) (Supplemental Material 2). Within-person comparisons showed that NRS scores were 0.89 points higher at time points when people had somatic/nociceptive pain compared with times without somatic/nociceptive pain (95% CI, 0.69–1.09; *P*<0.0001); between-person comparisons showed that the pain score was 0.87 points higher for a person with somatic/nociceptive pain than a person without somatic/nociceptive pain (95% CI, 0.67–1.08; *P*<0.0001). There were no significant associations between nociceptive/neuropathic pain and pain scores and dynamic pain (Supplemental Material 2).

### Linear mixed models analysis for oxycodone dose according to pain type

Between-person comparisons in the whole cohort showed that there was a small increase in opioid dose requirement of 1.04 mg (95% CI, 0.46–1.62; *P*<0.001) higher for people with affective pain compared with people without affective pain, after controlling for age, gravidity, parity, and mode of delivery. Similarly, but to a lesser extent (lower coefficients), other pain types were associated with increased opioid dose (Table 4). Postpartum pain intensity was associated with multiple different pain types, including affective, visceral/nociceptive, somatic/nociceptive, neuropathic, and nociceptive pain (Table 4).

**Table 4:** Unadjusted and adjusted relationship of pain types on oxycodone dose and pain intensity. ∗Models adjusted for maternal age, gravidity, parity, and delivery mode.

Type of pain	Oxycodone dose (unadjusted)		Oxycodone dose (adjusted)∗	
	Estimate	*P*-value	Estimate	*P*-value
Affective/evaluative	1.63	<0.001	1.02	0.001
Visceral/nociceptive	0.02	0.78	0.24	<0.001
Somatic/nociceptive	0.54	0.03	0.67	0.01
Dynamic/evoked	1.11	0.38	1.52	0.26
Neuropathic	0.07	0.72	0.11	0.57
Nociceptive	–0.16	0.01	–0.39	<0.001
Nociceptive, neuropathic, or both	–0.18	0.21	–0.19	0.22
	**Pain intensity (unadjusted)**		**Pain intensity (adjusted)**	

	**Estimate**	***P*-value**	**Estimate**	***P*-value**

Affective/evaluative	1.83	<0.001	1.79	<0.001
Visceral/nociceptive	0.08	0.001	0.11	<0.001
Somatic/nociceptive	0.92	<0.001	0.89	<0.001
Dynamic/evoked	0.53	0.30	0.46	0.40
Neuropathic	0.79	<0.001	0.76	<0.001
Nociceptive	–0.18	<0.001	–0.20	<0.001
Nociceptive, neuropathic, or both	0.06	0.32	–0.003	0.96

### Effects of opioid use disorder status on mixed models results for pain type and pain intensity scores

A history of OUD strengthened the relationship between pain type, pain intensity, and opioid dose. For oxycodone dose, neuropathic and nociceptive/neuropathic pain had significant interactions with history of OUD (Supplemental Material 2). For pain intensity, nociceptive, visceral/nociceptive, and affective/evaluative pain had significant interactions with history of OUD, with affective/evaluative pain showing strongest (highest coefficients) relationships (Supplemental Material 2). Affective pain was associated with NRS scores that were 0.93 points higher in people with OUD compared with people without (95% CI, 0.35–1.51; *P*=0.002). Among people with OUD, pain scores were 6.24 points higher for people with affective pain than without affective pain (95% CI, 0.041–10.58; *P*=0.04). For an individual person with OUD, time points when nociceptive/neuropathic pain were experienced was associated with oxycodone doses 1.56 mg higher (95% CI, –0.54 to 3.65; *P*=0.01). Among people with OUD, the expected oxycodone dose was 11.38 mg higher for people with nociceptive/neuropathic pain than without (95% CI, 2.13–17.53; *P* = 0.01) (Supplemental Material 2).

## Discussion

The key finding of our study is that postpartum pain is multidimensional both within- and between-people, characterised by different pain types and intensities, and that people with and without OUD experience postpartum pain differently. These findings are important because they suggest that tailored therapies should be investigated for patients with and without OUD. We found that pain type in the postpartum period is significantly associated with both pain intensity and inpatient opioid use, and that among people with OUD, affective (emotional) and nociceptive/neuropathic (cramping, incisional/skin or tissue injury) pain drive these relationships. Based on these data, and in alignment with the IASP, we suggest that future research on postpartum pain should focus on improving bedside clinical assessments of pain beyond the routine 0–10 numeric rating scale, and that future research focus on tailoring pain treatments depending upon the manifested pain type. For example, if the patient is describing pain that is primarily cramping, medications for cramping (such as NSAIDs) could be prioritised.

Our data also reveal quantifiable differences in pain experiences between people with and without OUD, for outcomes of inpatient opioid use and pain intensity scores, providing further direction for research on pain management in this special population of patients.

Whether after vaginal or Caesarean delivery, people with OUD experienced neuropathic and affective/evaluative pain more frequently than people without OUD. These distinctive postpartum pain experiences are potentially influenced by psychological or other complex social and biobehavioural interactions underlying a history of substance use disorders. Similar findings were also noted by Nidey and colleagues.^26^ For these reasons, pain in these patients may potentially be better managed by specific or a multimodal range of medication and non-medication options, although studies are required to assess this idea. The concept of multimodal analgesia in the acute perioperative setting typically refers to a combination of therapies, such as local anaesthesia or nerve blocks, and preoperative, perioperative, and postoperative acetaminophen, NSAIDs, or gabapentin,^27^^,^^28^ to reduce the need to use opioids for severe pain. Some non-opioid therapeutic options that require further investigation in this population and clinical context include cognitive–behavioural, complementary interventional, and physical-rehabilitation therapies. These non-opioid modalities have been shown to be effective in managing other types of pain^29^, ^30^, ^31^, ^32^ but require further research in postpartum obstetric pain, particularly for patients with OUD.

The tension between using and not using opioids for pain control particularly in patients with OUD warrants scrutiny. We found of a distinctive pattern of pain intensity and opioid use among patients with OUD. That finding highlights the importance of identifying right-sized therapies because for some people with OUD, the acute pain associated with labour and delivery can trigger return to use, whereas for others, targeted pulse doses of opioids may be completely appropriate for treatment of severe acute pain. Current efforts to curb opioid prescribing are emphasising modification of prescribing behaviours,^8^^,^^33^^,^^34^ but may overlook the importance of good pain control in reducing opioid use. Indeed, current pain management after birth may be insufficient for most people and is associated with restrictive opioid practices; one 2015 European multicentre cohort study^35^ revealed that Caesarean delivery pain management was insufficient and that the poor pain control was mainly associated with low opioid administration. Thus, focus on opioid prescribing behaviours alone in addressing the current opioid crisis may have unintended negative consequences on the successful management of postpartum pain and recovery for all people.

To our knowledge, this is the first study in postpartum patients to quantify pain type in detail and relate it to pain intensity and opioid use in hospital. We are also not aware of any study that has investigated the interaction of OUD on these relationships in other acute care settings, such as perioperative pain. Meyer and colleagues^36^^,^^37^ examined whether methadone or buprenorphine maintenance therapy for OUD changes labour pain or postpartum pain medication requirements. They found that neither of these medications changed analgesic needs and pain during labour, but that post-Caesarean delivery pain was increased in people who receive buprenorphine for maintenance therapy, and that a greater proportion of people who receive methadone or buprenorphine maintenance therapy require opioid analgesia compared with a control group (70% and 47%, respectively). Previous research has identified the challenge of managing pain during pregnancy and postpartum period in people with OUD and has called attention to the need for compassionate evidence-based care.^38^ Research has targeted reduced overall opioid consumption in postpartum patients,^8^^,^^9^^,^^39^ but has not focused on pain type and other nuanced aspects of pain.

Our finding that affective dimensions of pain are associated with increased oxycodone use requires additional research. It is possible that affective pain that is experienced more often and rated more intensely by patients with OUD is being treated appropriately with higher opioid doses on a short-term basis. Alternatively, patients with OUD who exhibit emotional or affective components of pain may be more likely trigger a recommendation for an opioid dose by the bedside nurse. We believe these findings require further probing, specifically focused on the role of affective pain as a driver for opioid dosing owing to the psychosocial aspects of pain, *vs* affective pain as a pathophysiologic mechanism, the risk of which is increased in patients with OUD. The optimal treatment for affective pain dimensions also requires additional study.

Similarly, our finding of visceral pain differences between mode of delivery is notable. Among patients without OUD, we observed higher rates of visceral pain after vaginal deliveries than Caesarean deliveries. Similar significant differences between visceral pain frequencies by mode of delivery were seen in patients with OUD. These observations may indicate that cramping pain from uterine involution is the predominant driver of the pain experience after a vaginal delivery, whereas other pain types – incisional, dynamic – become more contributory to the pain experience after Caesarean delivery. Altogether, these findings point to differential therapeutic options based on mode of delivery; vaginal delivery analgesia should emphasise visceral pain treatments (e.g. NSAIDs), whereas Caesarean deliveries should include these treatments but also incorporate treatment for other pain types (e.g. neuraxial morphine, supplemental local anaesthetics for nociceptive, neuropathic pain). It is worth noting that these differences in pain type frequencies between mode of delivery do not necessarily correlate with increased oxycodone dose and pain intensity. The results of our multilevel linear mixed effects modelling suggest that affective/evaluative, nociceptive, and neuropathic pain are key drivers of postpartum pain intensity and opioid dose.

Our study has limitations. Although the pain scores and descriptive words were provided directly by the patient, the bedside nurse asked and documented the ratings, rather than the patient selecting them. However, the ratings were provided by the patient without interpretation of the response by a clinician or anyone else.^40^ Coded pain terms were used rather than the actual pain inventories, perhaps leading to less comprehensive reporting. Pain intensity scores were asked throughout the shift at set 6-h intervals, but the scores did not account for the timing of administration of pain medications or patient activity. Thus, this study cannot speak to differences in response to pain treatment. Although we included mode of delivery as a covariate in the primary analysis, and although we did describe differences in pain types, intensity, and opioid use by mode of delivery, the primary analysis did not stratify by mode of delivery and thus cannot comment on distinct characteristics of pain type and their ramifications on intensity and opioid dose, according to mode of delivery. Our findings are consistent with many others in that mode of delivery drives pain intensity, and we also found patterns of pain type occur by mode of delivery which is consistent with clinical observations. The statistically significant differences identified in the current study may be of marginal clinical relevance (e.g. 1 mg differences in oxycodone dose). However, we submit that albeit small, the detected differences in this observational study can better point researchers in specific directions for additional investigations to replicate or explain why and how pain types drive opioid use behaviours or requirements. Also, this study did not distinguish between patients receiving buprenorphine or methadone. These medications have different effects on the mu-opioid receptor, which may affect pain experience, pain ratings, and opioid use. Future studies should compare these outcomes between patients receiving buprenorphine or methadone. Finally, we cannot account for other determinants of pain phenotype, such as presence of chronic back pain, psychiatric co-morbidities, fear of pain, and pain anxiety, among others. Patients with chronic back pain may have different pain outcomes and medication requirements that may not be reflected in this data set. These other psychiatric elements are not currently queried or documented in routine clinical care.

In conclusion, pain type is linked to postpartum opioid dose requirements. Affective dimensions of pain show the highest contributions to this relationship. For people with opioid use disorder, affective and nociceptive/neuropathic pain are key drivers of pain intensity and opioid consumption. Sensitive and multifaceted measurement tools that comprehensively assess the postpartum acute pain experience are needed, especially for people with opioid use disorder. Pain treatments for people with opioid use disorder must factor key differences in affective, nociceptive, and neuropathic pain to manage postpartum pain most effectively.

## Authors' contributions

Study design: GL, SC

Data abstraction: GL

Analysis: GL, KRL, BLR

Informed analysis: SC

Writing of the manuscript: all authors

Approved final version of the manuscript: all authors

## References

[1] Raja S.N., Carr D.B., Cohen M. (2020). The revised international association for the study of pain definition of pain: concepts, challenges, and compromises. Pain.

[2] Carvalho B., Butwick A.J. (2017). Postcesarean delivery analgesia. Best Pract Res Clin Anaesthesiol.

[3] Anderson T.A., Quaye A.N.A., Ward E.N., Wilens T.E., Hilliard P.E., Brummett C.M. (2017). To stop or not, that is the question: acute pain management for the patient on chronic buprenorphine. Anesthesiology.

[4] Leighton B.L., Crock L.W. (2017). Case series of successful postoperative pain management in buprenorphine maintenance therapy patients. Anesth Analg.

[5] Boltunova A., White R.S., Noori S., Chen S.A., Gaber-Baylis L.K., Weinberg R. (2019). Pre-existing opioid use disorder and postoperative outcomes after appendectomy or cholecystectomy: a multi-state analysis, 2007–2014. J Opioid Manag.

[6] Schiff D.M., Nielsen T., Terplan M. (2018). Fatal and nonfatal overdose among pregnant and postpartum women in Massachusetts. Obstet Gynecol.

[7] Smid M.C., Stone N.M., Baksh L. (2019). Pregnancy-associated death in Utah: contribution of drug-induced deaths. Obstet Gynecol.

[8] Prabhu M., McQuaid-Hanson E., Hopp S. (2017). A shared decision-making intervention to guide opioid prescribing after cesarean delivery. Obstet Gynecol.

[9] Prabhu M., Dubois H., James K. (2018). Implementation of a quality improvement initiative to decrease opioid prescribing after cesarean delivery. Obstet Gynecol.

[10] Ritchie C.S., Garrett S.B., Thompson N., Miaskowski C. (2020). Unintended consequences of opioid regulations in older adults with multiple chronic conditions. Gerontologist.

[11] Lam L., Richardson M.G., Zhao Z., Thampy M., Ha L., Osmundson S.S. (2021). Enhanced discharge counseling to reduce outpatient opioid use after cesarean delivery: a randomized clinical trial. Am J Obstet Gynecol MFM.

[12] Lakhi N., Tricorico G., Kanninen T., Suddle R., Ponterio J., Moretti M. (2019). Post-cesarean delivery outpatient opioid consumption and perception of pain control following implementation of a restrictive opioid prescription protocol. Am J Obstet Gynecol MFM.

[13] Mehdiratta J.E., Saab R., Chen Z., Li Y.J., Habib A.S. (2020). Patient and procedural risk factors for increased postoperative pain after cesarean delivery under neuraxial anesthesia: a retrospective study. Int J Obstet Anesth.

[14] Eisenach J.C., Pan P., Smiley R.M., Lavand'homme P., Landau R., Houle T.T. (2013). Resolution of pain after childbirth. Anesthesiology.

[15] Landau R., Bollag L., Ortner C. (2013). Chronic pain after childbirth. Int J Obstet Anesth.

[16] Vergara F., Sardi N.F., Pescador A.C. (2020). Contribution of mesolimbic dopamine and kappa opioid systems to the transition from acute to chronic pain. Neuropharmacology.

[17] Lee B., Zhao W., Yang K.C., Ahn Y.Y., Perry B.L. (2021). Systematic evaluation of state policy interventions targeting the us opioid epidemic, 2007–2018. JAMA Netw Open.

[18] Dworkin R.H., Turk D.C., Revicki D.A. (2009). Development and initial validation of an expanded and revised version of the Short-form McGill Pain Questionnaire (SF-MPQ-2). Pain.

[19] Cella D., Riley W., Stone A. (2010). The Patient-Reported Outcomes Measurement Information System (PROMIS) developed and tested its first wave of adult self-reported health outcome item banks: 2005–2008. J Clin Epidemiol.

[20] Nowinski C.J., Cella D., Revicki D. (2015). Development of the promis nociceptive pain scale. Qual Life Res.

[21] Askew R.L., Cook K.F., Keefe F.J. (2016). A PROMIS measure of neuropathic pain quality. Value Health.

[22] Schmidt R., Willis W. (2007). Encyclopedia of pain.

[23] Ende H.B., Landau R., Cole N.M. (2021). Labor prior to cesarean delivery associated with higher post-discharge opioid consumption. PLoS One.

[24] Kliethermes C., Blazek K., Ali K., Nijjar J.B., Kliethermes S., Guan X. (2017). Postoperative pain after single-site versus multiport hysterectomy. JSLS.

[25] Meghani S.H., Quinn R., Ashare R. (2021). Impact of cannabis use on least pain scores among African American and white patients with cancer pain: a moderation analysis. J Pain Res.

[26] Nidey N., Carnahan R., Carter K.D. (2020). Association of mood and anxiety disorders and opioid prescription patterns among postpartum women. Am J Addict.

[27] Bollag L., Lim G., Sultan P. (2021). Society for obstetric anesthesia and perinatology: consensus statement and recommendations for enhanced recovery after cesarean. Anesth Analg.

[28] Feldheiser A., Aziz O., Baldini G. (2016). Enhanced Recovery after Surgery (ERAS) for gastrointestinal surgery: Part 2. Consensus statement for anaesthesia practice. Acta Anaesthesiol Scand.

[29] Che X., Cash R., Ng S.K., Fitzgerald P., Fitzgibbon B.M. (2018). A systematic review of the processes underlying the main and the buffering effect of social support on the experience of pain. Clin J Pain.

[30] Koele R., Volker G., van Vree F., van Gestel M., Koke A., Vliet Vlieland T. (2014). Multidisciplinary rehabilitation for chronic widespread musculoskeletal pain: results from daily practice. Musculoskelet Care.

[31] Scascighini L., Toma V., Dober-Spielmann S., Sprott H. (2008). Multidisciplinary treatment for chronic pain: a systematic review of interventions and outcomes. Rheumatology (Oxford).

[32] Uchino B.N. (2009). Understanding the links between social support and physical health: a life-span perspective with emphasis on the separability of perceived and received support. Perspect Psychol Sci.

[33] Goldstick J.E., Guy G.P., Losby J.L., Baldwin G., Myers M., Bohnert A.S.B. (2021). Changes in initial opioid prescribing practices after the 2016 release of the CDC guideline for prescribing opioids for chronic pain. JAMA Netw Open.

[34] Olsen N., Eagan A., Romutis K., Terplan M., Martin C.E. (2020). Evaluation of a new departmental policy to decrease routine opioid prescribing after vaginal delivery. Am J Obstet Gynecol MFM.

[35] Marcus H., Gerbershagen H.J., Peelen L.M. (2015). Quality of pain treatment after caesarean section: results of a multicentre cohort study. Eur J Pain.

[36] Meyer M., Paranya G., Keefer Norris A., Howard D. (2010). Intrapartum and postpartum analgesia for women maintained on buprenorphine during pregnancy. Eur J Pain.

[37] Meyer M., Wagner K., Benvenuto A., Plante D., Howard D. (2007). Intrapartum and postpartum analgesia for women maintained on methadone during pregnancy. Obstet Gynecol.

[38] Brown H.L. (2020). Opioid management in pregnancy and postpartum. Obstet Gynecol Clin North Am.

[39] Gold S., Figueiro-Filho E., Agrawal S., Selk A. (2020). Reducing the number of opioids consumed after discharge following elective cesarean delivery: a randomized controlled trial. J Obstet Gynaecol Can.

[40] Deshpande P.R., Rajan S., Sudeepthi B.L., Abdul Nazir C.P. (2011). Patient-reported outcomes: a new era in clinical research. Perspect Clin Res.

