# Supplementary material for: Associations between postpartum pain type, pain intensity and opioid use in patients with and without opioid use disorder: a cross-sectional study
Source: Br J Anaesth. 2022 Nov 10;130(1):94–102. doi: 10.1016/j.bja.2022.09.029 (PMC9900726; doi:10.1016/j.bja.2022.09.029)
Supplement: Multimedia component 1 [file mmc1.docx]

**Maternal Diagnostic Codes**

| - Opioid dependence   - Defined as: medication listed (Subutex, suboxone, buprenorphine, buprenorphine-naloxone, methadone); diagnostic codes (see below)   - ICD-9:     - 304.00 OPIOID DEPENDENCE-UNSPECIFIED     - 304.01 OPIOID DEPENDENCE-CONTINUOUS     - 304.02 OPIOID DEPENDENCE-EPISODIC     - 304.03 OPIOID DEPENDENCE, IN REMISSION     - 304.70 OPIOID OTHER DEP-UNSPECIFIED     - 304.71 OPIOID OTHER DEP-CONTINUOUS     - 304.72 OPIOID OTHER DEP-EPISODIC     - 304.73 OPIOID OTHER DEP-IN REMISSION     - 305.50 OPIOID ABUSE-UNSPECIFIED     - 305.51 OPIOID ABUSE-CONTINUOUS     - 305.52 OPIOID ABUSE-EPISODIC     - 305.53 OPIOID ABUSE-IN REMISSION   - ICD-10:   - F11.1 Opioid abuse   - F11.10 …… uncomplicated   - F11.11 …… in remission   - F11.12 Opioid abuse with intoxication   - F11.120 …… uncomplicated   - F11.121 …… delirium   - F11.122 …… with perceptual disturbance   - F11.129 …… unspecified   - F11.14 …… with opioid-induced mood disorder   - F11.15 Opioid abuse with opioid-induced psychotic disorder   - F11.150 …… with delusions   - F11.151 …… with hallucinations   - F11.159 …… unspecified   - F11.18 Opioid abuse with other opioid-induced disorder   - F11.181 Opioid abuse with opioid-induced sexual dysfunction   - F11.182 Opioid abuse with opioid-induced sleep disorder   - F11.188 Opioid abuse with other opioid-induced disorder   - F11.19 …… with unspecified opioid-induced disorder   - F11.2 Opioid dependence   - F11.20 …… uncomplicated   - F11.21 …… in remission   - F11.22 Opioid dependence with intoxication   - F11.220 …… uncomplicated   - F11.221 …… delirium   - F11.222 …… with perceptual disturbance   - F11.229 …… unspecified   - F11.23 …… with withdrawal   - F11.24 …… with opioid-induced mood disorder   - F11.25 Opioid dependence with opioid-induced psychotic disorder   - F11.250 …… with delusions   - F11.251 …… with hallucinations   - F11.259 …… unspecified   - F11.28 Opioid dependence with other opioid-induced disorder   - F11.281 Opioid dependence with opioid-induced sexual dysfunction   - F11.282 Opioid dependence with opioid-induced sleep disorder   - F11.288 Opioid dependence with other opioid-induced disorder   - F11.29 …… with unspecified opioid-induced disorder   - F11.9 Opioid use, unspecified   - F11.90 …… uncomplicated   - F11.92 Opioid use, unspecified with intoxication   - F11.920 …… uncomplicated   - F11.921 …… delirium   - F11.922 …… with perceptual disturbance   - F11.929 …… unspecified   - F11.93 …… with withdrawal   - F11.94 …… with opioid-induced mood disorder   - F11.95 Opioid use, unspecified with opioid-induced psychotic disorder   - F11.950 …… with delusions   - F11.951 …… with hallucinations   - F11.959 …… unspecified   - F11.98 Opioid use, unspecified with other specified opioid-induced disorder   - F11.981 Opioid use, unspecified with opioid-induced sexual dysfunction   - F11.982 Opioid use, unspecified with opioid-induced sleep disorder   - F11.988 Opioid use, unspecified with other opioid-induced disorder   - F11.99 …… with unspecified opioid-induced disorder |
| --- |
