# Supplementary material for: Associations between postpartum pain type, pain intensity and opioid use in patients with and without opioid use disorder: a cross-sectional study
Source: Br J Anaesth. 2022 Nov 10;130(1):94–102. doi: 10.1016/j.bja.2022.09.029 (PMC9900726; doi:10.1016/j.bja.2022.09.029)
Supplement: Multimedia component 2 [file mmc2.docx]

**Table.** Results of mixed linear effects modeling for outcome of oxycodone dose.

|  | Mixed Linear Effects Model | | | | Between- and Within-Effects Model | | | | | |  |
| --- | --- | --- | --- | --- | --- | --- | --- | --- | --- | --- | --- |
| Pain Type | **Coefficient** | **Standard Error** | **95% CI** | ***P*-Value** | **Coefficient** | **Standard Error** | **95% CI** | ***P*-Value** | **Coefficient mn** | **Model *P*-value** | **Interpretation** |
| Dynamic | 1.57 | 1.34 | -1.05 to 4.19 | 0.24 | 1.20 | 1.38 | -1.50 to 3.90 | 0.38 | 6.73 | 0.25 | No significant association between dynamic pain and oxycodone dose. |
| Affective/Evaluative | 1.04 | 0.30 | 0.46 to 1.62 | <0.001 | 0.95 | 0.31 | 0.35 to 1.55 | 0.002 | 1.01 | 0.33 | Within-person: For an individual person, at time points when affective/evaluative pain were reported, oxycodone doses were approximately 1.04 higher than when affective/evaluative pain were not reported, controlling for covariates.  Between-persons: The between-and within-effects are not different. Therefore, the expected oxycodone dose is  1.04 higher for a person who had affective/evaluative pain compared to a person who did not have affective/evaluative pain, controlling for covariates. |
| Somatic/Nociceptive | 0.65 | 0.26 | 0.14 to 1.16 | 0.01 | 0.70 | 0.28 | 0.16 to 1.24 | 0.01 | -0.50 | 0.57 | Within-person: For an individual person, time points when somatic/nociceptive pain were reported were associated with  oxycodone doses that were approximately 0.65 higher than when somatic/nociceptive pain were not reported, controlling for covariates.  Between-persons: Between- and within-effects are not different. Therefore, the expected oxycodone dose is 0.65 higher for a person who had somatic/nociceptive pain compared to a person who did not have somatic/nociceptive pai, controlling for covariates. |
| Nociceptive | -0.32 | 0.06 | -0.44 to -0.19 | <0.001 | -0.48 | 0.07 | -0.62 to -0.35 | <0.001 | 0.93 | <0.001 | Within-person: For an individual person, time points when nociceptive pain were reported were associated with resulted in oxycodone doses that were approximately 0.48 lower than when she did not have nociceptive pain, controlling for  covariates.  Between-persons: The expected oxycodone dose is 0.44 (= 0.93 + -0.48). Therefore, the expected oxycodone dose is 0.44 higher for a person who had nociceptive pain compared to a person who did not have nociceptive pain, controlling for covariates. |
| Visceral/Nociceptive | 0.16 | 0.07 | 0.04 to 0.30 | 0.01 | 0.32 | 0.07 | 0.18 to 0.46 | <0.001 | -0.88 | <0.001 | Within-person: For an individual person, time points when visceral/nociceptive pain were reported were associated with oxycodone doses that were approximately 0.32 higher than when visceral/nociceptive pain were not reported,  controlling for covariates.  Between-persons: The expected oxycodone dose is 0.57 (=-0.88 + 0.32) lower for a person who had visceral/nociceptive pain compared to a person who did not have visceral/nociceptive pain, controlling for other covariates. |
|  |  |  |  |  |  |  |  |  |  |  |  |
| *No OUD Group* | **Mixed Linear Effects Model** | | | | **Between- and Within-Effects Model** | | | | | |  |
| Pain Type | **Coefficient** | **Standard Error** | **95% CI** | **P-Value** | **Coefficient** | **Standard Error** | **95% CI** | **P-Value** | **Coefficient mn** | **Model P-value** | **Interpretation** |
| Neuropathic | -0.33 | 0.21 | -0.75 to 0.08 | 0.12 | -0.72 | 0.23 | -1.16 to -0.28 | 0.001 | 3.47 | <0.001 | Within-person: For an individual person, time points when neuropathic pain were reported were associated with in oxycodone doses that were approximately 0.72 lower oxycodone dose than when neuropathic pain was not reported, controlling for other covariates.  Between-persons: The expected oxycodone dose is 2.75 (= 3.47 + -0.72) higher for a person who had neuropathic pain compared to a person who did not have neuropathic pain, controlling for other covariates. |
| Nociceptive/Neuro-pathic | -0.50 | 0.16 | -0.80 to -0.19 | 0.002 | -0.57 | 0.17 | -0.91 to -0.24 | 0.001 | 0.50 | 0.25 | Within-person: For an individual person, time points nociceptive and/or neuropathic pain were reported resulted in  oxycodone doses that were approximately 0.50 lower than when nociceptive and/or neuropathic pain were not reported, controlling for covariates.  Between-persons: Between- and within- are similar; therefore, the expected oxycodone dose is 0.50 lower for a person who had nociceptive and/or neuropathic pain compared to a person who did not have nociceptive and/or neuropathic pain, controlling for covariates. |
|  |  |  |  |  |  |  |  |  |  |  |  |
| *OUD Group* | **Mixed Linear Effects Model** | | | | **Between- and Within-Effects Model** | | | | | |  |
| Pain Type | **Coefficient** | **Standard Error** | **95% CI** | ***P*-Value** | **Coefficient** | **Standard Error** | **95% CI** | ***P*-Value** | **Coefficient mn** | **Model *P*-value** | **Interpretation** |
| Neuropathic | 1.74 | 0.92 | -0.06 to 3.54 | 0.06 | 1.57 | 0.96 | -0.31 to 3.45 | 0.10 | 2.15 | 0.52 | No significant association between neuropathic Pain and oxycodone dose. |
| Nociceptive/Neuro-pathic | 2.21 | 1.03 | 0.19 to 4.23 | 0.03 | 1.56 | 1.07 | -0.54 to 3.65 | 0.15 | 9.83 | 0.01 | Within-person: For an individual person, time points when nociceptive and/or neuropathic pain were reported were associated with oxycodone doses that were approximately 1.56 higher than when nociceptive and/or neuropathic pain were not reported, controlling for covariates.  Between-persons: The expected oxycodone dose is 11.38 (9.83 + 1.56) higher for a person who had nociceptive and/or neuropathic pain compared to a person who did not have nociceptive and/or neuropathic pain, controlling for covariates. |

CI, confidence interval; OUD, opioid use disorder.

**Table**. Results of mixed linear effects modeling for outcome of pain score.

|  | | | |  |  |  |  |  |  |  |  |
| --- | --- | --- | --- | --- | --- | --- | --- | --- | --- | --- | --- |
|  | **Mixed Linear Effects Model** | | | | **Between- and Within-Effects Model** | | | | | |  |
| Pain Type | **Coefficient** | **Standard Error** | **95% CI** | ***P*-Value** | **Coefficient** | **Standard Error** | **95% CI** | ***P*-Value** | **Coefficient mn** | **Model *P*-value** | **Interpretation** |
| Dynamic | 0.46 | 0.54 | -0.61 to 1.52 | 0.40 | 0.42 | 0.55 | -5.67 to 12.14 | 0.45 | 3.23 | 0.48 | No significant association between Dynamic Pain and pain scores. |
| Somatic/Nociceptive | 0.896 | 0.10 | 0.69 to 1.09 | <0.001 | 0.87 | 0.10 | 0.67 to 1.08 | <0.001 | 0.84 | 0.21 | Within-person: For an individual person, times when she had Somatic/Nociceptive pain resulted in  pain scores that were approximately 0.89 higher than when she did not have Somatic/Nociceptive pain, controlling for covariates.  Between-persons: Therefore, the expected pain score is 0.89 higher for a person who had Somatic/Nociceptive pain compared to a person who did not have Somatic/Nociceptive pain, controlling for covariates. |
| Neuropathic | 0.77 | 0.08 | 0.61 to 0.92 | <0.001 | 0.72 | 0.08 | 0.56 to 0.88 | <0.001 | 2.03 | <0.001 | Within-person: For an individual person, times when she had neuropathic pain resulted in pain scores that were approximately 0.72 higher than when she did not have neuropathic pain, controlling for other covariates.  Between-persons: The expected pain score is 2.75 (= 2.03 + 0.72) higher for a person who had neuropathic pain compared to a person who did not have neuropathic pain, controlling for covariates. |
| Nociceptive/Neuro-pathic | 0.004 | 0.06 | -0.12 to 0.13 | 0.95 | -0.01 | 0.06 | -0.13 to 0.12 | 0.90 | 0.39 | 0.28 | No significant association between nociceptive and/or neuropathic pain and pain scores. |
|  |  |  |  |  |  |  |  |  |  |  |  |
| *No OUD Group* | **Mixed Linear Effects Model** | | | | **Between- and Within-Effects Model** | | | | | |  |
| Pain Type | **Coefficient** | **Standard Error** | **95% CI** | ***P*-Value** | **Coefficient** | **Standard Error** | **95% CI** | ***P*-Value** | **Coefficient mn** | **Model *P*-value** | **Interpretation** |
| Nociceptive | -0.20 | 0.03 | -0.25 to -0.15 | <0.001 | -0.22 | 0.03 | -0.27 to -0.16 | <0.001 | 0.31 | 0.01 | Within-person: For an individual person, times when she had Nociceptive pain resulted in pain scores that were approximately 0.22 lower than when she did not have Nociceptive pain, controlling for other covariates.  Between-persons: The expected pain score is 0.09 (= 0.31 + -0.22) higher for a person who had Nociceptive pain compared to a person who did not have Nociceptive pain, controlling for other covariates. |
| Visceral/Nociceptive | 0.12 | 0.03 | 0.06 to 0.17 | <0.001 | 0.13 | 0.03 | 0.07 to 0.18 | <0.001 | -0.24 | 0.07 | Within-person: For an individual person, times when she had Visceral/Nociceptive pain resulted in pain scores that were approximately 0.12 higher than when she did not have Visceral/Nociceptive pain, controlling for covariates.  Between-persons: Since between-and within-effects are the same. Therefore, the expected pain score is 0.12 higher for a person who had Visceral/Nociceptive pain compared to a person who did not have Visceral/Nociceptive pain, controlling for covariates. |
| Affective/Evaluative | 1.93 | 0.13 | 1.68 to 2.18 | <0.001 | 1.81 | 0.13 | 1.56 to 2.06 | <0.001 | 7.18 | <0.001 | Within-person: For an individual person, times when she had Affective/Evaluative pain resulted in pain scores that were approximately 1.81 higher than when she did not have Affective/Evaluative pain, controlling for other covariates.  Between-persons: The expected pain score is 8.98 (= 7.18 + 1.81) higher for a person who had Affective/Evaluative pain compared to a person who did not have Affective/Evaluative pain, controlling for other covariates. |
|  |  |  |  |  |  |  |  |  |  |  |  |
| *OUD Group* | **Mixed Linear Effects Model** | | | | **Between- and Within-Effects Model** | | | | | |  |
| Pain Type | **Coefficient** | **Standard Error** | **95% CI** | ***P*-Value** | **Coefficient** | **Standard Error** | **95% CI** | ***P*-Value** | **Coefficient mn** | **Model *P*-value** | **Interpretation** |
| Nociceptive | 0.02 | 0.11 | -0.19 to 0.24 | 0.82 | 0.02 | 0.11 | -0.19 to 0.23 | 0.85 | 0.16 | 0.84 | No significant association between nociceptive pain and pain scores. |
| Visceral/Nociceptive | -0.16 | 0.11 | -0.38 to 0.05 | 0.14 | -0.16 | -0.16 | -0.37 to 0.06 | 0.16 | -0.27 | 0.75 | No significant association between visceral/nociceptive pain and pain scores. |
| Affective/Evaluative | 0.10 | 0.29 | 0.42 to 1.57 | 0.001 | 0.93 | 0.30 | 0.35 to 1.51 | 0.002 | 5.31 | 0.05 | Within-person: For an individual person, times when she had Affective/Evaluative pain resulted in  pain scores that were approximately 0.93 higher than when she did not have Affective/Evaluative  pain, controlling for other covariates.  Between-persons: The expected pain score is 6.24 (= 5.31 + 0.93) higher for a person who had  Affective/Evaluative pain compared to a person who did not have Affective/Evaluative pain, controlling for other covariates. |

CI, confidence interval; OUD, opioid use disorder.
